# Association of verbal and non-verbal theory of mind abilities with non-coding variants of *OXTR* in youth with autism spectrum disorder and typically developing individuals: a case-control study

**DOI:** 10.1186/s12888-023-05461-w

**Published:** 2024-01-08

**Authors:** Rana Ghamari, Mohammad Tahmaseb, Atiye Sarabi-Jamab, Seyed-Alireza Etesami, Azar Mohammadzadeh, Fatemeh Alizadeh, Mehdi Tehrani-Doost

**Affiliations:** 1 Department of Cellular and Molecular Biology, Faculty of Biological Sciences, Kharazmi University, Tehran, Iran; 2 Research Center for Cognitive and Behavioral Sciences, Roozbeh Psychiatry Hospital, Tehran University of Medical Sciences, Tehran, Iran; 3 Department of Genomic Psychiatry and Behavioral Genomics (DGPBG), School of Medicine, Roozbeh Hospital, Tehran University of Medical Sciences (TUMS), Tehran, Iran; 4 School of Cognitive Sciences, Institute for Research in Fundamental Sciences (IPM), Tehran, Iran; 5 Department of Cellular and Molecular Science, Roshd Azma Company, Alborz, Iran; 6 Department of Psychiatry, School of Medicine, Tehran University of Medical Sciences, Tehran, Iran

**Keywords:** Autism spectrum disorder, Theory of mind, Oxytocin receptor gene, rs2268498, rs53576

## Abstract

**Background:**

The ability to attribute mental states to others is called theory of mind (ToM) and is a substantial component of social cognition. This ability is abnormally developed in individuals with autism spectrum disorder (ASD). Several studies over the past decade have identified the oxytocin receptor gene (*OXTR*) and its variants as promising components for explaining the molecular mechanisms underlying Theory of Mind (ToM). The main aim of this study is to examine the association between rs2268498 and rs53576, two functional single nucleotide polymorphisms (SNPs), and verbal and non-verbal ToM in children and adolescents with ASD and a group of typically developing youth.

**Methods:**

The study involved 44 children and adolescents with high-functioning ASD aged 8 to 18 years old and 44 TD individuals who were matched on age and sex. In all participants, blood samples were collected and rs2268498 and rs53576 were genotyped. Happe’s Strange Stories test and the moving shapes paradigm were used to measure verbal and non-verbal ToM in all participants.

**Results:**

The results of permutation tests and logistic regression suggested that in TD group, rs2268498 AA carriers showed significant higher scores in variables representing verbal ToM (ToM stories and appropriateness score) whereas, in ASD group, rs53576 AA carriers exhibited significant better performance in parameters related to non-verbal ToM (ToM general rule and intentionality score). The results of hierarchical clustering in both groups support the findings by distinguishing between language-related and language-independent aspects of ToM.

**Conclusions:**

In the present study, we examined the association between rs2268498 and rs53576 and social functioning in individuals with ASD and TD group. We found preliminary evidence that rs2268498 and rs53576 are associated with ToM related abilities in healthy individuals as well as in autistic individuals. Accordingly, rs2268498 and rs53576 may play an important role in predicting ToM capabilities. It will be necessary to conduct further research to address the association of genetic variants with a deficit in ToM in individuals with ASD.

**Supplementary Information:**

The online version contains supplementary material available at 10.1186/s12888-023-05461-w.

## Background

As far as social evolution is concerned, Homo sapiens is almost universally acknowledged as a highly social species capable of complex aspects of social functioning [1]. Humans’ ability to perceive social cues, motivation to interpersonal interactions, and maintaining relationships with others distinguishes them from other species, even the most closely related ones like chimpanzees [2–5]. In order to achieve competency in social interaction, we need to have social cognition, social motivation, social awareness, and skills in maintaining relationship with others. Recently, social cognition has attracted more attention and been studied in greater depth due to its hierarchical and multidimensional nature [6]. As a basic component of social cognition, Theory of Mind (ToM) is noteworthy. According to the definition of the ToM, it is the ability to attribute others’ behaviors to their mental states which are based on social stimuli [7]. Impairment in ToM ability may result in neuropsychiatric symptoms, with autism spectrum disorder (ASD) standing out as a prominent example [8, 9]. According to the Diagnostic and Statistical Manual of Mental Disorders, fifth edition (DSM-5), ASD is characterized by repetitive behaviors/restricted interests, as well as social communication difficulties which reflects ToM dysfunction or “mindblindness” [8, 10].

While ASD is enigmatic in its etiology, there are several studies discovering endophenotypes relating to genetic markers of this disorder [11]. In this regard, family and twin studies as well as genome wide association studies (GWAS) of human social behavior and social cognition have helped to clarify the heritability of these traits and the contribution of genetics to their development [12–15]. In light of these findings, it has become increasingly apparent that oxytocin (OXT) plays a central role in many aspects of social behavior, including ToM which is impaired in some neuropsychiatric disorder including ASD [16–18]. OXT is a 9-amino acid neuropeptide which is mediated through the OXT receptor (*OXTR*) [19, 20]. Located on chromosome 3p25, the *OXTR* gene encodes the human *OXTR* protein-coupled receptor class I G, consisting of three introns and four exons. In addition to being primarily expressed in the reproductive system, *OXTR* expression can also be detected in several brain regions such as the frontal cortex, amygdala, hypothalamus, and olfactory nucleus [20–22]. Therefore, it is not surprising that several single nucleotide polymorphisms (SNPs) within the coding, noncoding, and regulatory regions of *OXTR* have been associated with social cognition deficits as seen in ASD. A significant association between rs2254298 and rs53576 with autism in families has been found in Chinese Han population [23], Also a significant association between rs2268493, rs2254298, and rs53576 and high-function autism in Caucasian families [24, 25], sheds light on the potential association between *OXTR* SNPs and ASD. Additionally, some studies have stepped further and studied the association of *OXTR* SNPs with social endophenotypes underlying the autistic symptoms. In this regard, Yang et al. (2018) reported a significant association between rs2254298 and social deficit in ASD, while Skusea et al. (2014) found that rs237887 was significantly associated with human social recognition skills among individuals with ASD as well as individuals with first-degree relatives with ASD [26, 27].

In the context of different *OXTR* SNPs, rs2268498 and rs53576 appear to be two of the most promising candidate SNPs putatively associated with individual differences in social functioning. rs2268498 is a regulatory SNP located on 2 KB upstream of *OXTR* in promoter region. In 2017, Reuter et al. identified the significant association of rs2268498 G allele (AG, GG) with higher expression of *OXTR* in human hippocamp tissue and HEK-293 cell line [28]. This study suggests that the rs2268498 genotype may contribute to the regulation of the OXT biological pathway by regulating the expression of *OXTR* in brain. Upon discovering this finding, further researches were conducted to determine whether the rs2268498 genotype was associated with social cognition manifestations. The effects of rs2268498 A allele on non-verbal social perception [29], facial emotion recognition [30] and empathic concern [31] are a few examples of how rs2268498 may influence social cognition. Furthermore, rs53576 is an intronic SNP which is located within intron 3. The location of rs53576 raises the possibility that rs53576 may also play a role in the regulation of *OXTR* expression. A significant association of rs53576 GG genotype with social auditory processing [32], different empathy domains in Asian and European populations [33], and higher attachment-related anxiety [34] support a possible association of rs53576 with social relationship.

With regard to the above studies, it appears that it has largely been neglected in attempts to examine higher levels and more complex characteristics of social cognition. One of the basic components of social cognition which has received relatively scant attention is ToM. To date, little research has been conducted on *OXTR* SNPs, including rs2268498 and rs53576, in association with some related aspects to ToM. For instance, little information is available regarding the association of rs53576 with the Reading Mind in the Eyes Test (RMET) and the false-belief task, both of which are known to be ToM tasks [35, 36]. However, the missing link, which has not been addressed as it should, is language. Given that language plays an influential role in the development of ToM, it could be considered to be an important factor. Therefore, ToM capabilities can be categorized based on its relation with language skills. As of yet, few studies have tried to discriminate ToM skills based on the usage of language. In this regard, verbal ToM abilities refer to ToM skills that directly engage higher level verbal/linguistic abilities. For example, according to the findings of Šimleša et al., in verbal ToM tasks, individuals must be able to comprehend a particular mental state by following narratives and vignettes or express others’ mental state by using true linguistic expressions [37, 38] In contrast, as Kobayashi et al. stated in their study, non-verbal ToM skills require minimum verbal demands to understand and express abstract subjects’ intentions [39]. As of yet, different cognitive tasks have been developed and measure different aspects and components of ToM skills. Some of them assess ToM verbally, whereas the others examine non-verbal ToM abilities. Among ToM tasks, Happé’s Strange Stories test is the most prominent test which measures verbal ToM in particular [38]. Moreover, although most of the cartoon-based ToM tasks are able to measure non-verbal ToM components, Frith- Happé animation task (also known as moving shapes paradigm) can assess both verbal and non-verbal components of ToM [40]. It should also be noted that Happé’s Strange Stories test and Frith- Happé animation task specialized to evaluate verbal and non-verbal ToM components in both children and adolescents with high-functioning autism and have been validated (normalized) in different languages including Persian [41, 42]. So, it seems that these two tasks can distinguish ToM abilities based on the usage of language in children and adolescents with high-function autism as well as typically developing children and adolescents. Besides, most previous researches have focused on the associations between rs2268498 and rs53576, as well as other *OXTR* SNPs, and social cognition features in typically developing populations not ASD people. Thus, further investigations are required to bridge the gap between ToM and the molecular underlying mechanisms from the perspective of verbal abilities in both typically developing and ASD individuals.

Hence, the purpose of this study is to examine whether rs2268498 and rs53576 can be used to model verbal and non-verbal ToM abilities as well as other aspects of social functioning among typically developing children and adolescents and those with high-functioning ASD.

## Methods

An overall total of 88 unrelated individuals participated in this study. Forty-four individuals with ASD and forty-four typically developing (TD) individuals were recruited in the study. High-functioning ASD individuals were patients at the outpatient clinic of Roozbeh Psychiatric Hospital during the last year. The medical records of these individuals were made available in full coordination with the dean of the hospital, the parents/caregivers of these people were contacted and the study was explained to them, and the ASD individuals whose parents/caregivers agreed to participate in the study were included in the study and underwent cognitive and genetic measurements. ASD group consisted of 12 females and 32 males between the ages of 8 and 18. In the ASD group, participants were diagnosed as having high-functioning autism spectrum disorder (mild to moderate ASD) by an expert child and adolescent psychiatrist at the Roozbeh psychiatry hospital outpatient clinic based on DSM-5 criteria. Individuals with ASD had no comorbid neuropsychiatric conditions (such as attention-deficit/hyperactivity disorder, bipolar disorder, depression, epilepsy, etc.), and Intelligence quotient (IQ) scores of 90 or higher at the time of participation. The TD participants were recruited through online advertisements. Parents of the TD group were informed of the study after announcing their willingness to participate. Participants in the study were those whose parents consented to their children participating. The TD group was composed of 18 females and 26 males of matched ages and sex. Parental reports indicated that the TD participants had no family or personal history of psychiatric conditions and a minimum IQ score of 90 was obtained by TD individuals. Participants in both the ASD and TD groups participated voluntarily, had Iranian ancestry, and at the time of the study did not have any diseases related to the immune system (cancer, autoimmune disorders, etc.).

## Procedure

After informed consent was obtained from parents, participants were guided to a quiet room between 9:00 AM and 11:00 AM in order to complete the tasks. Moving shapes paradigm and Happé’s Strange Stories tasks were selected as measures of non-verbal and verbal ToM, which were administered in a pseudorandom sequence. Each participant was administered Raven’s progressive matrix test in order to determine their IQ level after completing the ToM tasks. A psychiatric examination interview was conducted with their parents based on the last six- month behaviors of the participants. Following the cognitive/ behavioral assessments, further molecular tests were conducted. The research has been approved by the Tehran University of Medical Sciences Research Ethics Committee, which is in compliance with the Helsinki declaration (IR.KHU.REC.1399.021).

## Measurements

### Cognitive and behavioral measurements

In order to establish the diagnosis and severity of the symptoms parents of individuals with ASD were interviewed using the Childhood Autism Rating Scale (CARS). The CARS is comprised of 15 items that assesses ASD symptoms. Each item in CARS is scored between 1 (absence of abnormality) and 4 (severe abnormality). A cutoff point of 25.5 is considered to be indicative of a high-function ASD, and a score of 25.5 or higher is indicative of a preclinical diagnosis of mild ASD. All individuals in the ASD group achieved a CARS score of 25.5 or greater [43, 44].

An adapted version of the moving shapes paradigm (aka Frith-Happé animations) [45] and a formerly validated Happé’s Strange Stories test [46] were used to examine the non-verbal and verbal ToM abilities of all participants. Each task was displayed on a 17-inch monitor while the responses were recorded. For the verbal ToM assessment, three blocks of unlinked, human, and ToM stories from Happé’s Strange Stories test were utilized. Each block contains eight vignettes and each vignette is scored from 0 (irrelevant) to 2 (relevant/specific). A question was asked after each story about the characters’ utterances and the main context in which they appeared. After the task was completed, participants received three scores, ranging from 0 to 16. The higher scores of strange stories test the better performance of the participant. Non-verbal ToM was measured using the moving shapes paradigm. The paradigm contains three blocks of random, goal-oriented, and ToM animations. Each block includes four short silent animations with two colored triangles as characters. The participants should accurately convey the storyline and describe the triangle’s intention at the end of each animation. The following variables were derived from the participants’ responses: (1) general rule (GR) = Correctly describing the animation’s intentions for each block (Random, Goal-directed, ToM), (2) intentionality score (IN) = to determine the ToM level of sophistication in the used words (3) appropriateness score (AP) = to retell the storyline in the correct sequence. A validation study of the moving shapes paradigm provides more details about the task.

### rs2268498 and rs53576 genotyping

Genomic DNA was extracted from 0.5 ml of peripheral blood using Salting-out protocol. By UV-spectrophotometry, the quantity of extracted DNA was determined and the presence of a sharp and clear band, in line with the ladder one kb band, on 1% agarose gel electrophoresis gave an indication of DNA quality. DNA contamination with other chemical and biological components was also measured using the 260/280 and 260/230 ratios of absorbance. Both SNPs were genotyped utilizing restriction fragment length polymorphism polymerase chain reaction (PCR-RFLP) based on previous researches [47, 48]. To digest both rs2268498 and rs53576, BslI (catalog number: #ER1201) and BamHI (catalog number: #FD0054) restriction enzymes were respectively employed, and primers were designed and checked for specify leveraging primer-blast’s web-based platform (https://www.ncbi.nlm.nih.gov/tools/primer-blast/). In the case of rs2268498, primers (forward: 5’ TAGGCTGTCTCACGGGCTAC 3’, reverse: 5’ TCGGCCTCGAAAATTACAGA 3’) were designed to anneal the DNA template at 58°C during 35 PCR cycles. The 448 bp PCR product was digested overnight at 37°C and digested product was run on 2% agarose gel electrophoresis for genotyping (AA: 266 bp,131 bp, 52 bp/ AG: 266 bp, 229 bp, 131 bp, 52 bp, 36 bp/ GG: 229 bp, 131 bp, 52 bp, 36 bp). Initially, the annealing temperature of primers for rs53576 (forward: 5’ GCCCACCATGCTCTCCACATC 3’, reverse: 5’ GCTGGACTCAGGAGGAATAGGGAC 3’) was 58 °C [49]. After 35 cycles of PCR, we obtained a 340 bp PCR product. A 30-minute digestion at 37 °C was followed by 2% gel electrophoresis and genotyping (GG: 340 bp/ AA: 120 bp, 220 bp/ AG: 120 bp, 220 bp, 340 bp).

### Statistical analysis

Statistical analyses were performed using the R programming language version 4.2.2 (R Core Team, 2022) in the RStudio environment in accordance with a 95% confidence interval (CI). Basic R commands and dplyr (https://CRAN.R-project.org/package=dplyr), tidyverse (10.21105/joss.01686), vegan (https://CRAN.R-project.org/package=vegan), cluster (Maechler, M., Rousseeuw, P., Struyf, A., Hubert, M., Hornik, K.(2022). cluster: Cluster Analysis Basics and Extensions. R package version 2.1.4.), StatMatch (D’Orazio M (2022). _StatMatch: Statistical Matching or Data Fusion_. R package version 1.4.1, https://CRAN.R-project.org/package=StatMatch), factoextra (Kassambara A, Mundt F (2020). _factoextra: Extract and Visualize the Results of Multivariate Data Analyses_. R package version 1.0.7, https://CRAN.R-project.org/package=factoextra), and dendextend (Tal Galili (2015). dendextend: an R package for visualizing, adjusting, and comparing trees of hierarchical clustering. Bioinformatics. DOI: 10.1093/bioinformatics/btv428) packages were recruited in order to write statistical test codes. Additionally, the findings were visualized using the ggplot2 package (H. Wickham. ggplot2: Elegant Graphics for Data Analysis. Springer-Verlag New York, 2016.). Pearson’s chi-square test and Wilcoxon test were used to compare demographic data including sex and age between ASD and TD groups. The haplotype analysis of rs2268498 and rs53576 was carried out based on expectation-maximization (EM) algorithm using Haploview software version 4.2 in order to ensure that there was no physical linkage and linkage disequilibrium (LD) between rs2268498 and rs53576. Besides, the deviation of both rs2268498 and rs53576 from Hardy-Weinberg Equilibrium (HWE) was estimated by “genetics” package (Gregory Warnes, with contributions from Gregor Gorjanc, Friedrich Leisch and Michael Man. (2021). genetics: Population Genetics. R package version 1.3.8.1.3. https://CRAN.R-project.org/package=genetics) recruitment. With the use of the permutation test, rs2268498 and rs53576 associations with Happé’s Strange Stories test, moving shapes paradigm, and IQ score were investigated within and between groups. Furthermore, hierarchical clustering was employed in order to distinguish different aspects of ToM based on similarities and differences in parameters extracted from Happé’s Strange Stories test and moving shapes paradigm. As a final step, in order to compare feature detection abilities between SNPs (rs2268498 and rs53576) and hierarchical clustering, and to investigate the relation between cognitive parameters and both rs2268498 and rs53576, a general linear model (GLM) with logistic regression was used. Besides, for each equation in GLM, odds ratio $\left( {{\text{OR}}\,{\text{ = }}\,{\text{log}}\left( {\frac{p}{{1 - p}}} \right)} \right)$ and *p* was calculated. It is important to note that, except for the within-group analysis, all statistical tests involving both ASD and TD groups used regression out method to adjust the effect of IQ.

## Results

### Demographic results

Pearson’s chi-square test indicated that there were no significant differences between the two groups based on gender (p = 0.260). Additionally, Wilcoxon test results did not reveal significant differences in age between individuals with ASD and TD group (p = 0.391). However, the IQ of the TD group was significantly higher than that of the ASD group, despite none of the participants attending exceptional schools (p = 1.883 × 10^− 4^). Therefore, IQ is controlled by regressing it out. A summary of the demographic results can be found in supplementary material 1 Table 1.

### rs2268498/rs53576 HWE and haplotype analysis

There was no significant deviation from HWE for rs2268498 and rs53576 based on Pearson’s chi-square test (p _rs2268498_ = 0.519, p _rs53576_ = 0.358). Additionally, rs2268498 and rs53576 do not exhibit a strong linkage based on the statistics and parameters obtained from haplotype analysis. In this respect, it could be considered that these two SNPs may act independently of one another. (D’ = 0.436, LOD = 3.070, r^2^ = 0.143). An overview of the HWE and genotype distribution is provided in supplementary material 1 Table 2.

### rs2268498/rs53576 and IQ score assessments

Meanwhile, it has been indicated that IQ score was significantly associated with rs53576 genotype across ASD and TD individuals (p-value = 0.028). Further analysis revealed that while there was no significant association between rs53576 genotype and IQ score in TD participants (p-value = 0.647), in ASD group individuals with rs53576 GG genotype had significantly lower IQ scores than A carriers (p-value = 0.023).

### rs2268498/rs53576 and ToM assessments

The differences between ASD and TD group in performing ToM tasks (Happé’s Strange Stories test and moving shapes paradigm) have been indicated in our previous finding [50]. In brief, there was no significant differences between ASD and TD group in performing unlinked (p = 0.841) and human stories (p = 0.784) blocks of Happé’s Strange Stories test as well as random animations GR of moving shapes paradigm (p = 0.853). However, the results revealed that in ToM stories blocks of Happé’s Strange Stories test (p ≤ 0.001) and other moving shapes paradigms components including Goal-directed GR (p ≤ 0.001), ToM GR (p ≤ 0.001), IN (p ≤ 0.001), and AP (p ≤ 0.001), ASD group have significant lower scores than TD group. These findings implicitly revealed that ASD individuals could follow the tasks’ instructions as well as TD individuals.

In total sample, Happé’s Strange Stories task results revealed a significant association of rs2268498 with ToM stories, but not with human or unlinked stories (p _ToM_ = 0.003, p _human_ = 0.056, p _unlinked_ = 0.883) which was replicated in both TD and ASD groups separately (p _ToM−ASD_ = 0.018, p _ToM−TD_ = 0.010). In the TD group, the rs2268498 AA genotype was significantly associated with higher scores in Happé’s Strange Stories task human and ToM blocks (p _ToM−TD_ = 0.005, p _human_ = 0.027), while in the ASD group, the rs2268498 AA genotype was significantly associated with higher performance only in the ToM block (p _ToM−ASD_ = 0.019).

**Fig. 1:**
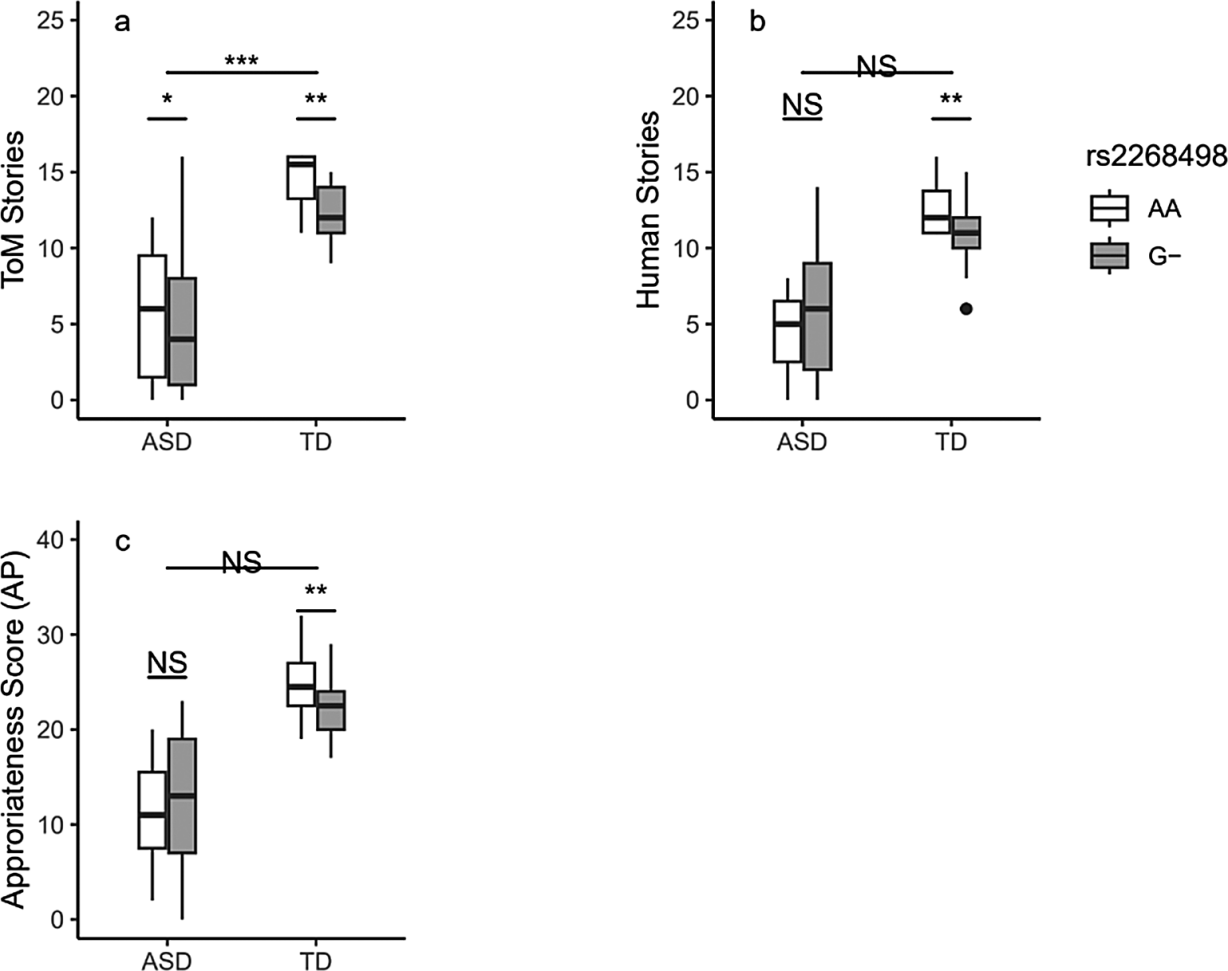
Association of rs2268498 with verbal ToM in both groups. NS refers to non-significant. (*): p-value ≤ 0.05, (**): p-value ≤ 0.01, (***): p-value ≤ 0.001

In rs53576, there was a significant association between rs53576 and ToM stories block in the total sample population (p _ToM_ ≤ 0.001, p _human_ = 0.192, p _unlinked_ = 0.527) but not in each group separately (p _ToM−ASD_ = 0.081, p _ToM−TD_ = 0.094). It shows that this significant difference arises from differences in ToM stories residual scores between individuals with ASD and TD individuals. Table 1; Fig. 1a & 1b provide detailed information regarding the results.

**Table 1:** Association analysis of rs2268498/rs53576 × ASD/TD with Happé’s strange stories test variables

SNP	Variable	Mean (± SD)		Df	Sum of Squares	R^2^	F	p-value*
		ASD	TD					
rs2268498	Unlinked stories	6.886 ± 3.828	9.977 ± 2.592	1	0.004	9.800 × 10^− 4^	0.093	0.833
	Human stories	5.386 ± 4.166	11.659 ± 2.010	1	0.173	0.039	3.816	0.057
	ToM stories	5 ± 4.393	13.204 ± 2.097	1	0.364	0.083	8.014	**0.003**
	Residual			84	3.819	0.875		
	Total			87	4.361	1.000		
rs53576	Unlinked stories	6.886 ± 3.828	9.977 ± 2.592	1	0.016	0.003	0.463	0.527
	Human stories	5.386 ± 4.166	11.659 ± 2.010	1	0.058	0.012	1.611	0.196
	ToM stories	5 ± 4.393	13.204 ± 2.097	1	1.619	0.342	44.821	**≤ 0.001**
	Residual			84	3.034	0.641		
	Total			87	4.728	1.000		

SD = standard deviation, df = degree of freedom

*Note*: Significant p-values (p-values which are less than 0.05 with 95% CI are highlighted in bold)

The results of the moving shapes paradigm indicated that in total samples, rs2268498 was significantly associated with ToM and goal-directed GR, as well as IN, but not with random animations GR and AP (p _ToM_ ≤ 0.001, P _goal−directed_ = 0.002, p _IN_ ≤ 0.001, p _random_ = 0.339, p _AP_ = 0.070). In within groups analysis, rs2268498 was only marginally associated with AP in the TD group (p _ToM−ASD_ = 0.647, P _goal−directed−ASD_ = 0.175, p _random−ASD_ = 0.109, p _ToM−TD_ = 0.215, P _goal−directed−TD_ = 0.289, p _random−TD_ = 0.437, p _IN−ASD_ = 0.444, p _IN−TD_ = 0.627, p _AP−TD_ = 0.047). Further analysis showed that rs2268498 G-carriers scored significantly lower in the AP variable than AA carriers in the TD group (p = 0.032).

**Fig. 2:**
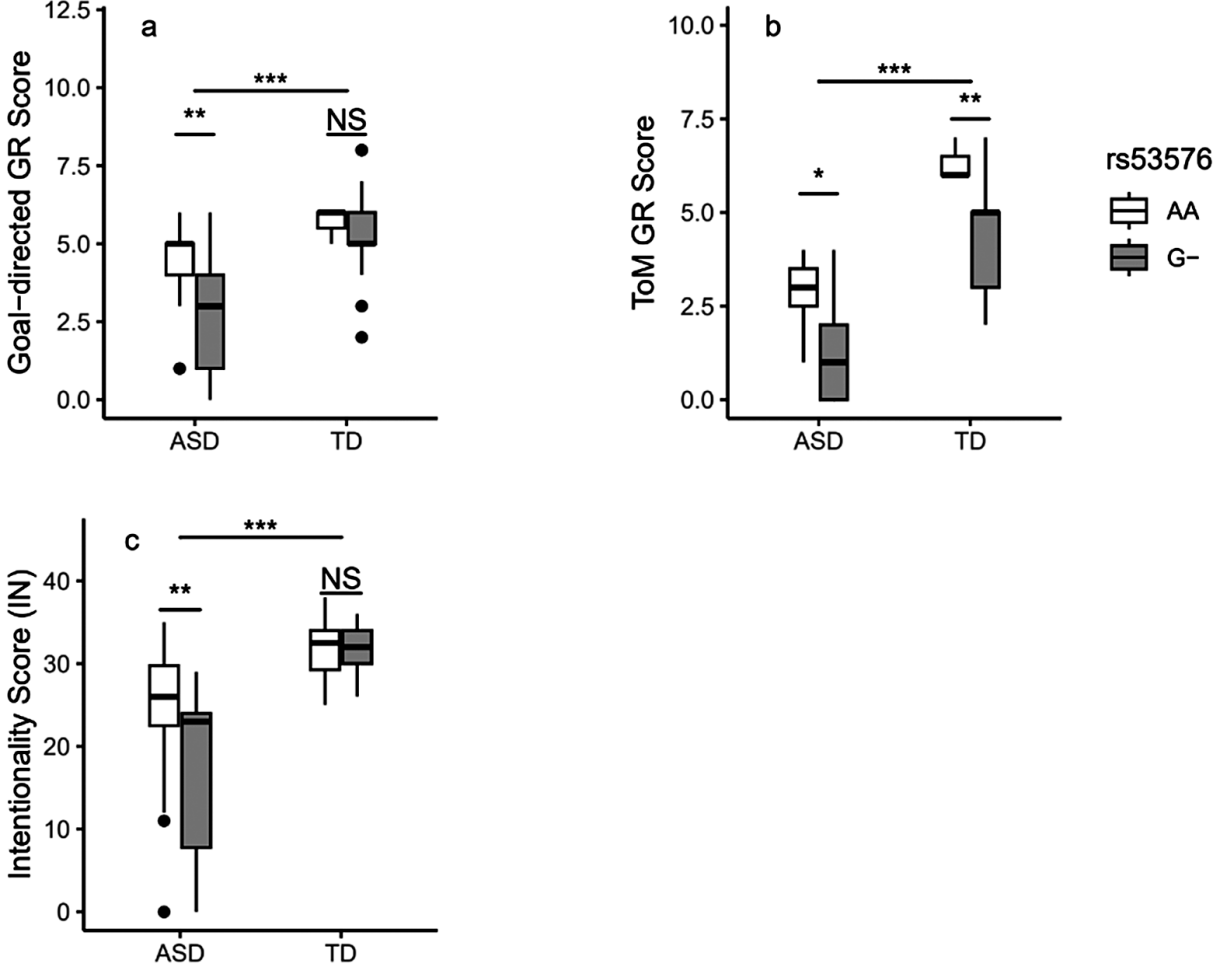
Association of rs53576 with non-verbal ToM in both groups

For rs53576, it was indicated that rs53576 was significantly associated with goal-directed and ToM animations GRs, IN, and AP but not random animations GR in the total sample (p _ToM_ = 0.002, P _goal−directed_ = 0.004, p _IN_ ≤ 0.001, p _AP_ = 0.031, p _random GR_ = 0.370). Within group analysis indicated that rs53576 was significantly associated with the goal-directed animations and the ToM animations GRs and IN scores in the ASD group but not in the TD group (p _ToM−ASD_ = 0.039, P _goal−directed−ASD_ = 0.006, p _random−ASD_ = 0.279, p _AP−ASD_ = 0.698, p _IN−ASD_ = 0.013, p _ToM−TD_ = 0.356, P _goal−directed−TD_ = 0.695, p _random−TD_ = 0.402, p _AP−TD_ = 0.571, p _IN−TD_ = 0.265). Additional investigations revealed that in the ASD group, individuals with rs53576 AA genotype scored significantly higher in goal-directed and ToM animations GRs (p _ToM_ = 0.039, P _goal−directed_ = 0.027, p _random_ = 0.636), while carriers of rs53576 GG genotype scored higher in IN variable but not AP (p AP = 0.310, p IN = 0.014). Figs. 1c, 2a, 2b, 2c and Table 2 provide additional statistics.

**Table 2:** Association of moving shapes paradigm variables’ residuals with “rs53576/rs2268498 × grouping”

SNP	Variable	Mean (± SD)		df	Sum of Squares	R^2^	F	p-value
		ASD	TD					
rs2268498	GR – random animations	3.045 ± 2.079	2.977 ± 1.355	1	0.041	0.009	1.057	0.339
	GR – goal-directed animations	2.954 ± 1.952	5.431 ± 1.189	1	0.490	0.112	12.457	**0.002**
	GR – ToM animations	1.568 ± 1.420	4.750 ± 1.366	1	0.520	0.119	13.199	≤ 0.001
	Residual			83	3.309	0.758		
	Total			87	4.361	1.000		
	IN	22.636 ± 9.594	31.977 ± 3.015	1	0.602	0.138	14.009	**≤ 0.001**
	AP	11.977 ± 6.428	23.454 ± 3.406	1	0.134	0.030	3.135	0.070
	Residual			85	3.610	0.827		
	Total			87	4.361	1.000		
rs53576	GR – random animations	3.045 ± 2.079	2.977 ± 1.355	1	0.040	0.008	0.882	0.370
	GR – goal-directed animations	2.954 ± 1.952	5.431 ± 1.189	1	0.419	0.088	9.176	**0.002**
	GR – ToM animations	1.568 ± 1.420	4.750 ± 1.366	1	0.431	0.091	9.456	**0.004**
	Residual			83	3.837	0.811		
	Total			87	4.728	1.000		
	IN	22.636 ± 9.594	31.977 ± 3.015	1	0.159	0.033	3.730	**0.031**
	AP	11.977 ± 6.428	23.454 ± 3.406	1	0.970	0.205	22.708	**≤ 0.001**
	Residual			85	3.590	0.759		
	Total			87	4.728	1.000		

*Note*: Significant p-values (p-values which are less than 0.05 with 95% CI are highlighted in bold)

### Hierarchical clustering results

**Fig. 3:**
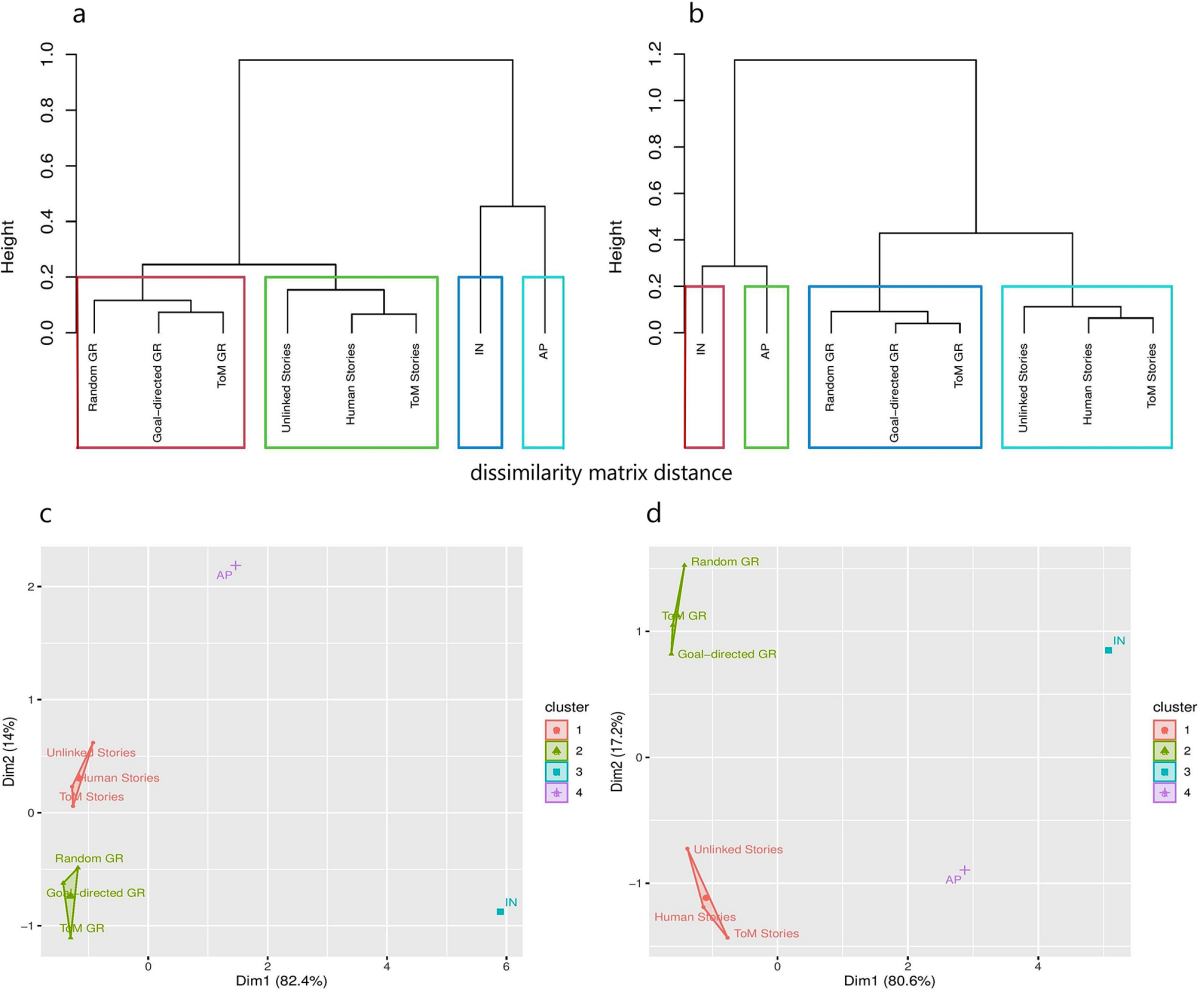
Hierarchical clustering results visualization. Hierarchical clustering of ToM parameters’ dissimilarity matrix distance in TD (**a**) and ASD (**b**) groups and 2-dimentional k-means clustering in TD (**c**) and ASD (**d**) groups

Using hierarchical clustering of dissimilarity matrix distances and for both ASD and TD groups, four clusters of cognitive parameters were found. According to the dendrograms, the cognitive variables were classified into four clusters based on the distance matrix: (1) General non-verbal characteristics regardless of ToM sophistication (random, goal-directed, and ToM GR), (2) The ability to consume verbally regardless of ToM sophistication (unlinked, human, and ToM stories), (3) The capability of acquiring non-linguistic ToM (IN), and (4) The ability to acquire verbal ToM (AP). Fig. 3a and 3b present ASD and TD clustering results. As a result of visualizing 2-dimensional K-means clustering, it was revealed that in the TD group, the parameters representing similar ToM features (verbal/non-verbal) have a relatively smaller distance from one another than other variables. Meanwhile, the parameters illustrating verbal and non-verbal ToM were visually farther apart from each other in the ASD and TD groups. Fig. 3c and 3d illustrate 2-dimensional clustering in more detail.

### General linear model and logistic regression

None of the ToM parameters in the ASD group was predicted by the rs2268498 AA genotype. However, in the TD group, the rs2268498 AA genotype was significantly associated with AP, human, and ToM stories, which are partially associated with verbal ToM and verbal skills (β_human_ = 1.423, p _human_ = 0.019, OR _human_ = 4.150; β_ToM_ = 2.286, p _ToM_ = 1.440 × 10^− 4^, OR _ToM_ = 9.838; β_AP_ = 2.333, p _AP_ = 0.023, OR _AP_ = 10.312). Using GLM results for rs53576 in both groups, it was found that in ASD group, rs53576 AA genotype significantly predicted GR of goal-directed and ToM animations, and GG genotype significantly predicted IN responses (β_goal−directed_ = 1.583, p _goal−directed_ = 0.047, OR _goal−directed_ = 4.870; β_ToM_ = 1.532, p _ToM_ = 7.270 × 10^− 3^, OR _ToM_ = 4.631; β _IN_ = -7.324, p _IN_ = 0.016, OR _IN_ = 6.596 × 10^− 4^). The rs53576 AA genotype was also found to be a significant predictor of ToM animations GR in individuals who were TD (β_ToM_ = 1.699, p _ToM_ = 0.035, OR _ToM_ = 5.470). There is a detailed presentation of GLM results in supplementary material 1 Table 3.

## Discussion

In this study, we examined the association of two *OXTR* non-coding SNPs, rs2268498 and rs53576, with verbal and non-verbal ToM abilities and model ToM skills in ASD and TD participants independently. Based on the convergence of results from permutation tests and GLM analysis, it was determined that rs2268498 AA genotype was significantly associated with verbal ToM in the TD group, whereas rs53576 AA genotype was significantly associated with non-verbal ToM in the ASD group. Moreover, it has been suggested that rs53576 was significantly associated with IQ score in ASD patients.

This finding could be attributed to the evolutionary role that *OXT* and *OXTR* play in the social perception of vertebrates, particularly mammals. In 2022, it was demonstrated that homozygous deletion of the *Oxtr* could result in social isolation in eight-week-old zebrafish [51]. Also, earlier in 2016, it was suggested that *OXTR* expression changes in the Nucleus Accumbens (NAcc) play a significant role in the regulation of social behavior in prairie voles. According to the results of this study, there are several non-coding polymorphisms in the *OXTR* regulatory and intronic regions that are associated with altered *OXTR* expression in the NAcc [52]. This may lend support to the contention that *OXTR* plays a significant role in social perception in mammals.

As discussed earlier, due to the complexity of social cognition processes in humans, *OXTR* has a much broader and more complex impact on humans than it does on other species. As a result, the effects of *OXT* and *OXTR* variants can be observed in humans from prenatal development to adulthood, as well as in daily social interactions seen in individuals with social cognition impairment such as ASD. As an example of the effect of *OXTR* variation during infancy, it was reported in 2015 by Unternaehrer et al. that maternal behaviors in childhood have a significant impact on *OXTR* methylation pattern during adulthood. The study discovered that individuals who received less maternal care during childhood exhibit significantly higher methylation of *OXTR* during adulthood, which is significantly associated with the incidence of psychiatric disorders [53].

Particularly in the case of ASD, several studies tried to shed light on the association of *OXTR* with ASD and its related phenotypes. The association of SNPs linked to *OXTR* with ASD likelihood according to meta-analyses results [54], the correlation of genetic and epigenetic modifications of *OXTR* with aberrant social behavior [55], the association of ASD clinical features (e.g. seizures, panic, and aggressive behaviors) with *OXTR* SNPs [56], the association of rs53576 with social, emotional or behavioral functioning in children and adolescents [57], and the relation of *OXTR* polymorphisms with social impairment in children with and without ASD [58] are illustrations of studies that find significant associations of *OXTR* variants with ASD and its correlated traits.

The results of this study (association of rs53576 with non-verbal ToM performance in ASD group) are largely explained by this previous evidence. While different studies have demonstrated the association between various variants of *OXTR* and maternal behavior effects on toddlers, rs2268498 and rs53576 stand out in this field due to their significant interaction with environmental factors both during childhood and adulthood. In 2019, Smarius et al. suggested that five-to-six-year-old children who had the GG genotype in rs53576 and were exposed to verbal violence by their mothers, as well as same age children who were carriers of the A allele in rs2268498 (AA and AG) and were exposed to verbal violence by their mothers, showed a significant increase in systolic blood pressure [59]. In addition, Sicorello et al. in 2020 attempted to identify associations between rs2268498 and rs53576 and everyday social behaviors, such as social buffering, among healthy individuals. Their results demonstrated that G carriers of both rs2268498 and rs53576 exhibited lower social company requirements after stressful events [60]. In light of the findings discussed above, it is implied that rs2268498 and rs53576 together and alongside each other may be significantly associated with social functioning. As a result, further investigation may be needed in order to gain a deeper understanding of the mechanisms behind the association of rs2268498 and rs53576 with social cognition. Regarding this approach, Laursen et al. investigated the association between rs2268498 and rs53576 polymorphisms and cognitive empathy in 2014. The results indicated that healthy individuals who hold the CC and AA genotypes at rs2268498 and rs53576, respectively, displayed significantly higher levels of empathic concern [61]. Moreover, in 2019, Meixner et al. assessed if rs2268498 and rs53576 are associated with language-based ToM through the use of a word-processing task. The results illustrated that rs2268498 A carriers and rs53576 G carriers showed weaker bias in conditions measuring emotionally positive self-related words [62]. Thus, it could be anticipated that rs2268498 and rs53576 may also be associated with verbal ToM on the basis of such an observation. In this respect, our findings demonstrate a positive association between the rs2268498 AA genotype and validated parameters pertaining to language-based ToM abilities in typically developing children and adolescents. However, in the case of the association of rs53576 with IQ our findings suggest the association of rs53576 GG genotype with lower IQ scores in ASD individuals. This seems contradictory to some extent [49]. Hence, this should be evaluated in other samples and population for replicability. In this regard, it is worth mentioning that a number of previous studies have also examined the association of *OXTR* polymorphisms and validated ToM tasks. Hence, some studies have attempted to determine if there is a significant association between *OXTR* SNPs and well-known ToM tasks such as the Reading Mind in the Eyes’ Test (RMET) and the false-belief task. The obtained results showed no significant association between *OXTR* SNPs and false-belief task performance in healthy populations regardless of age [36, 63], whereas RMET accuracy in healthy adolescents and young adults was significantly associated with rs53576, rs2254298, and rs2228485 [35]. While rs2268498 has been shown to be significantly associated with autistic traits in healthy young adults [64], no study has yet been able to find an association between *OXTR* variants and ToM tasks such as RMET in population with ASD symptoms [65]. Moreover, considering that individuals with ASD may also present language delays, it may be more appropriate to use language-related and language-free ToM tasks separately. In this regard, our results clearly illuminated that rs53576 is associated with non-verbal ToM in individuals with ASD. Despite of findings regarding association between rs53576 and rs2268498 with different aspects of ToM, including this study, the accuracy and reliability of these findings are still in question. In light of this, it appears that these results require assistance on a computational level. Using the matrix distance principle, we have found that linguistic and nonlinguistic ToM differ from one another in both ASD and TD individuals. According to our results, rs53576 was associated with parameters that represent verbal ToM in the ASD group, while rs2268498 was associated with variables indicative of verbal ToM in the TD group.

ToM can have various manifestations in everyday life. These manifestations can range from following social cues during infancy to complex abilities intertwined with other cognitive functions, such as language and face processing. Both rs2268498 and rs53576 have indicated significant associations with different aspects of ToM or of which ToM itself is a part (e.g., empathy, face emotion recognition, social cognition, etc.). However, the question that arises before deep diving into the discussion about the association of rs2268498 and rs53576 with these aspects of ToM is that considering the fact that these two variants are located in the non-coding regions of the *OXTR* gene, how and through what mechanisms they have association with ToM? Regarding rs2268498, since this SNP is located in the promoter region of *OXTR* gene and has a significant association with *OXTR* expression at the cellular level [28], it can be concluded that the association of rs2268498 with ToM is moderated by *OXTR* expression. Different studies have uncovered the association between rs2268498 and ToM. Previous findings suggested that rs2268498 has a significant association with identifying and utilizing social cues as well as social learning which in turn can represent ToM [29, 66]. In addition, rs2268498 genotype can predict prosocial behavior and empathic concerns, both indicators of ToM [31]. More importantly, rs2268498 illustrated association with facial emotion recognition, an ability that directly reflects ToM associated functions [30].

rs53576 is a non-coding SNP located in intron 3. Up to now, no study has revealed the exact cellular and molecular mechanisms through which rs53576 affects ToM. However, it can be assumed that rs53576 could play a role as a cis-SNP and regulate *OXTR* expression itself or be a trans-SNP for other genes associated with ToM. As of yet, a wide range of investigations have tried to demonstrate the association of rs53576 with ToM and its associated traits. In infants, rs53576 has a significant association with visual attention to the eyes as social cues [67]. Additionally, it has been shown that rs53576 is significantly associated with RMET performance [35] and Hinting Task [68] which both are ToM measuring tasks in typically developing individuals. ToM has, however, been largely correlated with empathy and rs53576 in previous studies. While Gong et al. demonstrated the association of rs53576 with empathy in general [69], the study of Chander et al. revealed that rs53576 GG genotype is associated with greater cognitive empathy in Asian cohorts [33]. Moreover, McDonald et al. studies, rs53576 moderates the relation of parent-child interactions with children’s empathy [70]. Sociality, sociability, social cognition abilities, face processing, and facial emotion recognition are other manifestations of ToM associated with rs53576 [71–74]. rs53576 is not just associated with ToM in typically developing populations. Some studies have indicated the association of rs53576 with ToM in individuals with psychiatric symptoms. As an illustration, rs53576 is significantly associated with ASD [75], Asperger syndrome [76], and autism related social impairment and social communication difficulties [58, 77]. Furthermore, rs53576 has also shown a significant correlation with ToM in individuals with treatment-resistant schizophrenia [78], bipolar disorder type I [79], and attention deficit/hyperactivity disorder (ADHD) [80]. Lastly, few studies evaluate the association of rs53576 and ToM at the neural level. Association of rs53576 with N1 event-related potential component during performing a social task [81] and correlation of anterior cingulate and supplementary motor area activity with rs53576 during performing an empathic task [82], are studies indicating the relation of rs53576 and ToM at neurobiological level.

Despite all the studies mentioned above, no study distinguished verbal and non-verbal ToM variations using rs2268498 and rs53576 across high-functioning autistic and TD individuals. There is only one study in 2019 that evaluates the association between rs2268498 and rs53576 with emotional words related to self and others [62]. Therefore, this study is the first evaluation to discriminate verbal ToM components from non-verbal ToM using rs2268498 and rs53576 across ASD and TD individuals, and the results have implicitly been validated by hierarchical clustering.

While many aspects have been discussed so far, the most pressing question here is through which neurobiological mechanisms *OXTR* affects cognitive and behavioral functions related to ToM? There are two levels of explanation for these questions: cellular and neurobiological. At the cellular level, there is scarce evidence for *OXTR* function in neurons. The general consensus is that the *OXT* and *OXTR* signaling pathways in neurons are similar to those in uterine smooth muscle cells. Only in 2022, Meyer et al. showed that in HEK293 cell lines, mutation in *OXTR* (A218T) leads to *OXTR* protein stability, shift in Ca2 + dynamics, which ends in MAPK pathway activation reduction [83]. Meanwhile, it is generally accepted that neurobiological changes bridge the gap between cellular modifications associated with *OXTR* genetic variants and ToM characteristics. In 2014, Brian W. Haas et al. published one of the first studies in this field. The results of this study indicated that *OXTR* is significantly associated with social endophenotypes. These endophenotypes include brain regions such as dorsolateral prefrontal cortex (DLPFC), Ventromedial Prefrontal Cortex (VMPFC), Visual Cortex (VC), Premotor Cortex (PMC), and Anterior Cingulate Cortex (ACC), which are essential for various social cognition processes, including emotion recognition and social reward, social communication, empathy, and ToM. Researchers hypothesized that *OXTR* is associated with social traits indirectly by affecting these brain regions [84]. Thus, when it comes to rs53576, homozygote individuals with GG genotype who experienced insecure childhood attachments have shown higher brain gray matter volumes in the left amygdala and lower volumes in the right superior parietal lobule, left temporal pole, and bilateral frontal regions during the ToM paradigm [34]. In addition, Uzefovsky et al. in 2019 found significant association between rs53576 GG genotype and hyperactivity in the right supramarginal gyrus (rSMG) and inferior parietal lobule (rIPL) during RMET (as a ToM task) whereas AA genotype was associated with topological patterns in brain functional networks [85, 86]. Nevertheless, little is known about the association between rs2268498 and brain scale endophenotypes. For instance, Zimmermann et al. showed that rs2268498 AA is associated with Amygdala functional connectivity [87]. Considering these findings together, they shed light on the association between rs2268498 AA genotype and rs53576 GG genotype and changes in brain regions related to ToM. In this sense, our findings at the cognitive level were implicitly aligned with the results of those studies.

The main limitation of this study was the sample size. Due to limitations in budget, time, facilities, and time overlap with COVID-19 pandemic we could not expand the sample size enough. However, using accurate statistical methodologies (e.g., permutation tests) we could overcome this limitation to some extent. Further, advanced assessments using neuroimaging techniques and assays that measure other DNA variants with larger sample size are required to evaluate the accuracy of these findings.

## Conclusions

In conclusion, here, children and adolescents with ASD and typically developing individuals, matched on sex and age in the Iranian population were assessed for verbal and non-verbal ToM as well as *OXTR* two variants, rs2268498 and rs53576, respectively. It was discovered that rs2268498 could significantly predict verbal ToM of TD participants, whereas rs53576 could significantly predict non-verbal ToM of participants with ASD. Additionally, the genetic and hierarchical modeling results aligned with each other and showed partial agreement. In order to ensure that the results of this study are reliable, further examinations will be required.

### Electronic supplementary material

Below is the link to the electronic supplementary material.

**Supplementary Material 1: Table 1.** Demographic results of participants. **Table 2.** Genotype distribution and HWE results of rs2268498 and rs53576

## Data Availability

The dataset supporting the conclusions of this article is available in G-Node GIN repository (https://gin.g-node.org/ranaghamari/OXTR).

## List of abbreviations

ACC: Anterior Cingulate Cortex
AP: appropriateness score
ASD: Autism Spectrum Disorder
CARS: Childhood Autism Rating Scale
CI: Confidence Interval
DSM-5: Diagnostic and Statistical Manual of Mental Disorders, fifth edition
DLPFC: dorsolateral prefrontal cortex
GR: general rule
GWAS: Genome Wide Association Study
HWE: Hardy-Weinberg Equilibrium
IQ: Intelligence quotient
IN: Intentionality score
LD: linkage disequilibrium
NAcc: Nucleus Accumbens
OXT: oxytocin
*OXTR*: Oxytocin Receptor gene
PMC: Premotor Cortex
RMET: Reading Mind in the Eyes Test
SNP: single nucleotide polymorphism
ToM: Theory of Mind
TD: Typically developing
VMPFC: Ventromedial Prefrontal Cortex
VC: Visual Cortex
