# Supplementary material for: Association of verbal and non-verbal theory of mind abilities with non-coding variants of OXTR in youth with autism spectrum disorder and typically developing individuals: a case-control study
Source: BMC Psychiatry. 2024 Jan 8;24:30. doi: 10.1186/s12888-023-05461-w (PMC10773038; doi:10.1186/s12888-023-05461-w)
Supplement: Supplementary file 1 — Supplementary Material 1: Table 1. Demographic results of participants. Table 2. Genotype distribution and HWE results of rs2268498 and rs53576 [file 12888_2023_5461_MOESM1_ESM.docx]

# **Supplementary Material 1**

|  | **Gender** | | | | | **Age** | | | |
| --- | --- | --- | --- | --- | --- | --- | --- | --- | --- |
| **Group** | **Type** | **Frequency** | **Chi -squared** | **df** | **p-value** | **Mean** | **SD** | **W** | **p-value** |
| **ASD** | **Female** | 12 (27%) | 1.264 | 1 | 0.260 | 12.159 | 2.884 | 1070.500 | 0.391 |
|  | **Male** | 32 (73%) |  |  |  |  |  |  |  |
| **TD** | **Female** | 18 (40%) |  |  |  | 11.590 | 2.404 |  |  |
|  | **Male** | 26 (60%) |  |  |  |  |  |  |  |

Supplementary Table 1: Demographic results of participants

|  | **rs2268498** | | | **HWE p-value** | **rs53576** | | | **HWE p-value** |
| --- | --- | --- | --- | --- | --- | --- | --- | --- |
|  | **AA** | **AG** | **GG** |  | **AA** | **AG** | **GG** |  |
| **ASD** | 11 (25%) | 21 (48%) | 12 (27%) | 0.772 | 7 (16%) | 23 (52%) | 14 (32%) | 0.519 |
| **TD** | 18 (41%) | 19 (43%) | 7 (16%) | 0.752 | 3 (7%) | 23 (52%) | 18 (41%) | 0.160 |
| **Total** | 29 (33%) | 40 (45%) | 19 (22%) | 0.519 | 10 (11%) | 46 (52%) | 32 (37%) | 0.358 |

Supplementary Table 2: genotype distribution and HWE results of rs2268498 and rs53576

| **Parameters** | **ASD** | | | | | | **TD** | | | | | |
| --- | --- | --- | --- | --- | --- | --- | --- | --- | --- | --- | --- | --- |
|  | **Coefficients** | **Standard Error** | **t value** | **p-value** | **OR** | **P** | **Coefficients** | **Standard Error** | **t value** | **p-value** | **OR** | **P** |
| **Intercept** | 4.242 | 2.043 | 2.076 | 0.044 | 1.833 | 0.647 | 9.982 | 0.816 | 12.234 | 1.970 × 10^-15^ | 9.838 | 0.907 |
| **ToM stories ~**  **rs2268496** | 0.606 | 1.544 | 0.392 | 0.696 |  |  | 2.286 | 0.546 | 4.181 | **1.440 × 10^-4^** |  |  |
| **Intercept** | 5.690 | 2.008 | 2.833 | 0.007 | 0.592 | 0.371 | 11.440 | 0.927 | 12.337 | 1.490 × 10^-15^ | 3.497 | 0.777 |
| **ToM stories ~ rs53576** | -0.523 | 1.436 | -0.365 | 0.717 |  |  | 1.252 | 0.621 | 2.015 | 0.0503 |  |  |
| **Intercept** | 6.939 | 1.925 | 3.605 | 8.220 × 10^-4^ | 0.288 | 0.223 | 9.653 | 0.871 | 11.077 | 4.850 × 10^-14^ | 4.150 | 0.805 |
| **Human stories ~ rs2268496** | -1.242 | 1.455 | -0.854 | 0.398 |  |  | 1.423 | 0.584 | 2.437 | **0.019** |  |  |
| **Intercept** | 7.376 | 1.880 | 3.924 | 3.170 × 10^-4^ | 0.221 | 0.180 | 10.051 | 0.893 | 11.252 | 2.950 × 10^-14^ | 3.130 | 0.757 |
| **Human stories ~**  **rs53576** | -1.510 | 1.344 | -1.123 | 0.267 |  |  | 1.141 | 0.598 | 1.906 | 0.0635 |  |  |
| **Intercept** | 7.454 | 1.782 | 4.183 | 1.430 × 10^-4^ | 0.634 | 0.388 | 8.730 | 1.182 | 7.381 | 4.170 × 10^-9^ | 2.422 | 0.707 |
| **Unlinked stories ~ rs2268496** | -0.454 | 1.347 | -0.337 | 0.737 |  |  | 0.884 | 0.792 | 1.116 | 0.271 |  |  |
| **Intercept** | 8.738 | 1.727 | 5.060 | 8.750 × 10^-6^ | 0.245 | 0.196 | 7.935 | 1.153 | 6.883 | 2.140 × 10^-8^ | 4.257 | 0.809 |
| **Unlinked stories ~ rs53576** | -1.405 | 1.235 | -1.137 | 0.262 |  |  | 1.448 | 0.772 | 1.875 | 0.067 |  |  |
| **Intercept** | 1.757 | 0.945 | 1.858 | 0.070 | 2.801 | 0.736 | 3.320 | 0.625 | 5.313 | 3.830 × 10^-6^ | 0.783 | 0.440 |
| **Random GR ~**  **rs2268496** | 1.030 | 0.715 | 1.441 | 0.157 |  |  | -0.243 | 0.418 | -0.582 | 0.564 |  |  |
| **Intercept** | 1.890 | 0.933 | 2.026 | 0.049 | 2.401 | 0.705 | 2.658 | 0.625 | 4.251 | 1.160 × 10^-4^ | 1.254 | 0.556 |
| **Random GR ~**  **rs53576** | 0.876 | 0.667 | 1.313 | 0.196 |  |  | 0.226 | 0.419 | 0.541 | 0.591 |  |  |
| **Intercept** | 3.939 | 0.895 | 4.399 | 7.290 × 10^-5^ | 0.454 | 0.312 | 4.739 | 0.538 | 8.796 | 4.470 × 10^-11^ | 1.634 | 0.620 |
| **Goal-directed GR ~ rs2268496** | -0.787 | 0.677 | -1.164 | 0.251 |  |  | 0.491 | 0.361 | 1.361 | 0.181 |  |  |
| **Intercept** | 1.119 | 0.944 | 1.186 | 0.242 | 4.870 | 0.830 | 5.162 | 0.788 | 6.546 | 6.500 × 10^-8^ | 1.286 | 0.562 |
| **Goal-directed GR ~ rs53576** | 1.583 | 0.776 | 2.038 | **0.047** |  |  | 0.252 | 0.718 | 0.351 | 0.728 |  |  |
| **Intercept** | 2.060 | 0.657 | 3.135 | 0.003 | 0.674 | 0.402 | 4.683 | 0.632 | 7.406 | 3.840 × 10^-9^ | 1.048 | 0.511 |
| **ToM GR ~ rs2268496** | -0.393 | 0.496 | -0.793 | 0.432 |  |  | 0.047 | 0.423 | 0.111 | 0.912 |  |  |
| **Intercept** | -0.208 | 0.660 | -0.316 | 0.753 | 4.631 | 0.822 | 2.935 | 0.860 | 3.411 | 0.001 | 5.470 | 0.845 |
| **ToM GR ~ rs53576** | 1.532 | 0.543 | 2.822 | **0.007** |  |  | 1.699 | 0.784 | 2.167 | **0.035** |  |  |
| **Intercept** | 24.758 | 4.458 | 5.554 | 1.740 × 10^-6^ | 0.183 | 0.154 | 32.188 | 1.395 | 23.060 | 2.000 × 10^-16^ | 0.861 | 0.462 |
| **IN ~ rs2268496** | -1.697 | 3.370 | -0.504 | 0.617 |  |  | -0.149 | 0.935 | -0.160 | 0.874 |  |  |
| **Intercept** | 32.290 | 4.099 | 7.878 | 8.290 × 10^-10^ | 6.596 × 10^-4^ | 8.483 × 10^-5^ | 32.055 | 1.396 | 22.960 | 2.000 × 10^-16^ | 0.945 | 0.485 |
| **IN ~ rs53576** | -7.324 | 2.932 | -2.498 | **0.016** |  |  | -0.055 | 0.935 | -0.059 | 0.953 |  |  |
| **Intercept** | 13.455 | 2.986 | 4.506 | 5.200 × 10^-5^ | 0.306 | 0.234 | 20.166 | 1.482 | 13.600 | 2.000 × 10^-16^ | 0.103 | 0.093 |
| **AP ~ rs2268496** | -1.182 | 2.257 | -0.524 | 0.603 |  |  | 2.333 | 0.993 | 2.348 | **0.023** |  |  |
| **Intercept** | 13.867 | 2.927 | 4.737 | 2.490 × 10^-5^ | 0.238 | 0.192 | 21.756 | 1.553 | 14.013 | 2.000 × 10^-16^ | 3.337 | 0.769 |
| **AP ~ rs53576** | -1.433 | 2.094 | -0.685 | 0.497 |  |  | 1.205 | 1.040 | 1.158 | 0.253 |  |  |

Supplementary Table 3: GLM results; rs2268498/rs53576 are independent variables and cognitive parameters are dependent variables
